# Positive and negative correlates of social media use, including aggression, trauma and suicide

**DOI:** 10.1192/j.eurpsy.2023.139

**Published:** 2023-07-19

**Authors:** E. Sönmez Güngör

**Affiliations:** Psychiatry, Erenköy Mental Health and Neurological Diseases Training and Research Hospital, Istanbul, Türkiye

## Abstract

**Abstract:**

In the past two decades, social media has become the central way for many people, organizations, institutions to communicate and share opinions, ideas, and information. A young individual on average spends 2-3 hours on social media, engaging in 6-7 applications, which has also dramatically increased during/and after the pandemic. So far, there has been increasing evidence that social media influences behavior, including its own use. It has also been related with aggression towards self and others, and especially suicidal behaviour related (positively and negatively) with social media use has received growing academic and public interest. Excessive use of social media or addiction, exposure to negative content, stalking, cyberbullying and cybervictimization are among concepts which have an influence on (self-)aggressive behavior. Research has shown that adolescents are more vulnerable to cybervictimization than adults, and female adolescents, who spend more time on social media than males, are even more vulnerable, resulting in depressive and in some cases, suicidal ideation. Individuals in marginalized groups might also be more prone to be affected by excluding discourse or discriminatory posts. On the other hand, social media could serve as a coping mechanism given the possibilities for instant support from online networks, availability of psychoeducational resources and mental health related scientific content. Few meta-analyses in the field indicate the need to systematize sampling and data collection; while each pointing to the heterogeneity of the impact of social media. In this talk, the correlates of social media will be reviewed, and possible mental health-promoting approaches will be discussed.

**Disclosure of Interest:**

None Declared

